# Prognostic factors for survival after curative resection of gastric mixed adenoneuroendocrine carcinoma: a series of 80 patients

**DOI:** 10.1186/s12885-018-4943-z

**Published:** 2018-10-22

**Authors:** Jian-Wei Xie, Jun Lu, Jia-Bin Wang, Jian-Xian Lin, Qi-Yue Chen, Long-Long Cao, Mi Lin, Ru-Hong Tu, Ze-Ning Huang, Ju-Li Lin, Chao-Hui Zheng, Ping Li, Chang-Ming Huang

**Affiliations:** 1 Department of Gastric Surgery, Fujian Medical University Union Hospital, No. 29 Xinquan Road, Fuzhou, 350001 Fujian Province China; 2 Department of General Surgery, Fujian Medical University Union Hospital, Fuzhou, China; 3 Key Laboratory of Ministry of Education of Gastrointestinal Cancer, Fujian Medical University, Fuzhou, China; 4 Fujian Key Laboratory of Tumor Microbiology, Fujian Medical University, Fuzhou, China

**Keywords:** Stomach neoplasms, Mixed adenoneuroendocrine carcinoma, Prognosis, Recurrence, Risk factors

## Abstract

**Background:**

To assess the prognostic factors and investigate the optimal treatment of gastric mixed adenoneuroendocrine tumors.

**Methods:**

We retrospectively analyzed clinical data from 80 patients with gastric mixed adenoneuroendocrine carcinoma that received radical resection in our department from January 2007 to December 2016. Risk factors for relapse and survival were analyzed using a multivariate Cox proportional hazards regression model. Gastric mixed adenoneuroendocrine carcinoma was divided into neuroendocrine carcinoma and adenocarcinoma based on the predominant type in the tumor.

**Results:**

The 3-year overall survival was 40% in the neuroendocrine carcinoma group and 75% in the adenocarcinoma group (*P* = 0.006). The neuroendocrine carcinoma (NEC)-dominant tumors and a Ki-67-positive index ≥60% were independent risk factors for worse overall survival. The 3-year recurrence-free survival was 33% in the neuroendocrine carcinoma group and 68% in the adenocarcinoma group. NEC-dominant tumors and a Ki-67-positive index ≥60% were independent risk factors for gastric mixed adenoneuroendocrine carcinoma recurrence. Patients in the adenocarcinoma group that received adjuvant chemotherapy exhibited significantly better overall survival than patients that did not receive chemotherapy (median survival time 43 months vs. 13 months, *P* = 0.026).

**Conclusion:**

The NEC-dominant tumors and a Ki-67-positive index ≥60% were significantly associated with worse survival and a higher recurrence rate for gastric mixed adenoneuroendocrine carcinoma patients. Patients in the adenocarcinoma group may benefit from gastric adenocarcinoma treatments.

**Electronic supplementary material:**

The online version of this article (10.1186/s12885-018-4943-z) contains supplementary material, which is available to authorized users.

## Background

Gastric neuroendocrine tumors (gNETs) are rare neoplasms with significant heterogeneity that account for approximately 4.0% of all neuroendocrine tumors [1], and the incidence of gNET increases yearly [2, 3]. According to the 2010 WHO classification, gNET can be divided into neuroendocrine tumors, neuroendocrine carcinomas and mixed neuroendocrine cancers [3]. Gastric mixed adenoneuroendocrine carcinomas (gMANECs) contain gastric adenocarcinoma and neuroendocrine carcinoma cells, with each population accounting for at least 30% of tumor cells [4]. The pathology of gMANEC indicates that its clinical and biological characteristics differ from those of simple gastric neuroendocrine carcinoma and simple gastric adenocarcinoma [5]. However, due to the low incidence of gMANEC, few gMANEC studies have been performed, most of which analyze only a single or a few patients [6–8]. In addition, no guidelines currently exist for gMANEC patients, as the 2017 National Comprehensive Cancer Network guidelines do not address gMANEC treatment, and factors affecting gMANEC prognosis and treatment have not yet been reported. Therefore, we retrospectively investigated the clinicopathologic data of gMANEC after radical gastrectomy in a large-volume center to analyze prognostic factors and identify appropriate adjuvant treatments.

## Methods

### Patient selection

We retrospectively analyzed clinical data from patients diagnosed with gMANEC at Union Hospital, Fujian Medical University, from January 2007 to December 2016. The inclusion criteria were as follows: (1) those pathologically diagnosed with gMANEC; (2) those without distant metastasis, as assessed by preoperative examination; and (3) those who underwent D2 lymph node dissection and for whom postoperative pathological diagnosis of R0 resection was conducted. The following were the exclusion criteria: (1) those with preoperative and intraoperative findings of distant metastasis; (2) those who underwent preoperative adjuvant chemotherapy or radiotherapy; and (3) those with incomplete clinical data. A total of 80 gMANEC patients were included in this study. The study was approved by the ethics committee of the Fujian Medical University Union Hospital. Written consent was provided by the patients for their information and specimens to be stored in the hospital database and used in research.

### Diagnosis and classification of gMANEC

gMANEC was defined as a malignant tumor containing a proportion of at least 30% or more of both glandular epithelial cells and neuroendocrine cells according to the 2010 classification of neuroendocrine tumors. All neuroendocrine tumors were confirmed, diagnosed and classified using microscopic histomorphological features, immunohistochemical staining for neuroendocrine tumor-associated biomarkers. The pathological findings were confirmed by two experienced pathologists.

**Fig. 1:**
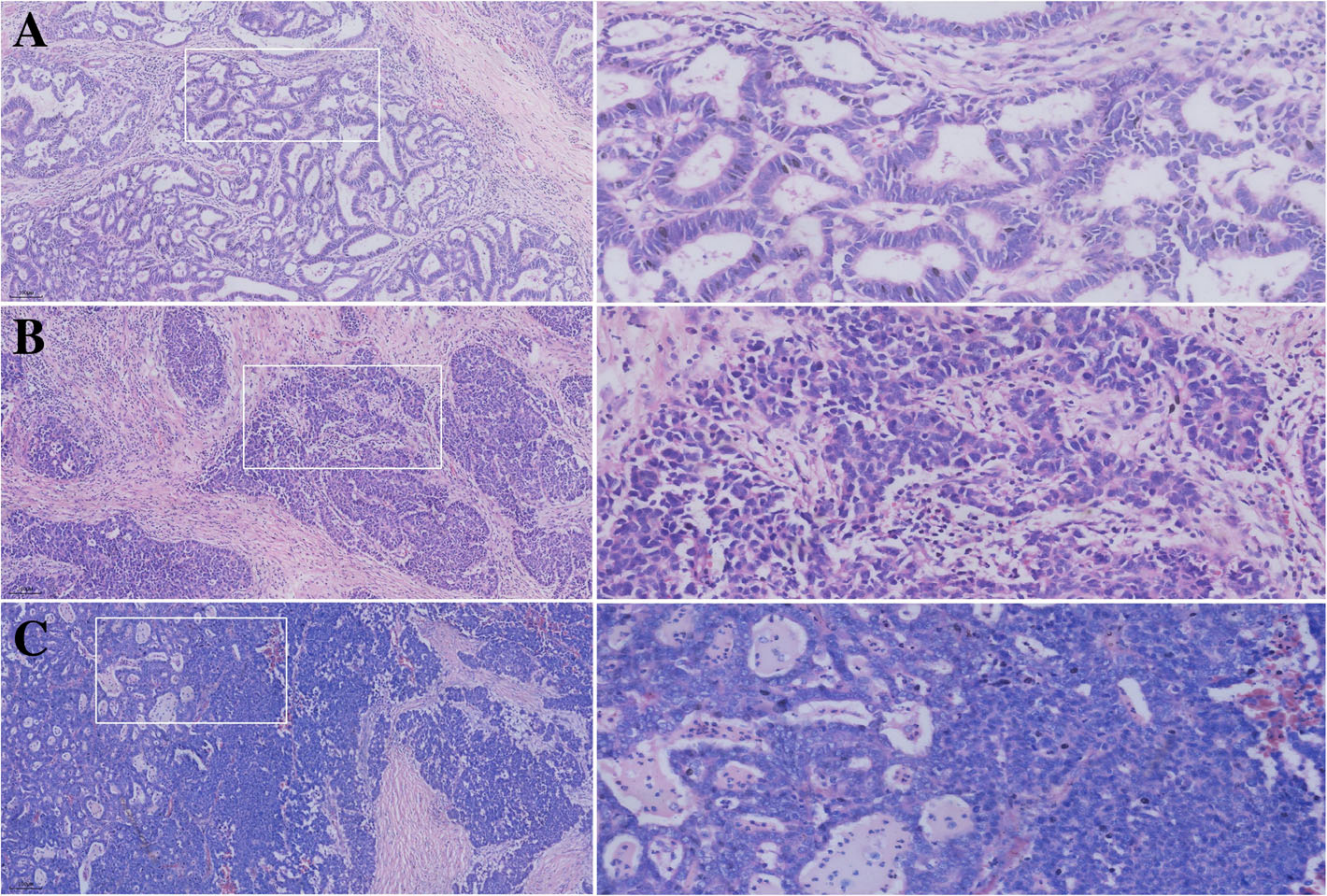
Different components in lymph node metastasis. (**a**) adenocarcinoma component. (**b**) neuroendocrine carcinoma component. (**c**) mixed components with neuroendocrine carcinoma and adenocarcinoma

Tumor dominance: When the tumor had not metastasized, the tumor component in the primary tumor was greater than 50% of all tumors. If lymph node metastasis occurred, the number of metastatic lymph nodes in the tumor component was greater than 50% of all metastatic lymph nodes. gMANEC was divided into the neuroendocrine carcinoma (NEC) group and the adenocarcinoma (AC) group according to the type of tumor dominance: NEC-dominant type (Fig. 1a) and AC-dominant type (Fig. 1b).

### Variables and definitions

Overall survival (OS) was recorded from the time of surgery to the last follow-up, date of death or the deadline of the follow-up database (such as lost to follow-up or other causes of death). Recurrence-free survival was the time from the first diagnosis to the initial recurrence. Post-recurrence survival was the time from the initial recurrence to the date of death. The following definitions for recurrent tumors were utilized: (1) local recurrence - gastric stump or anastomotic recurrence, metastasis to lymph nodes around the stomach; (2) hematogenous metastasis - metastasis to remote organs (e.g., the liver, lung, brain, bone) or to periaortic lymph nodes or lymph nodes outside the abdominal cavity; and (3) peritoneal recurrence - a tumor derived from the peritoneum or ovary [9]. A tumor size of 5 cm was considered the cut-off point. For Ki-67, 60% positive was considered the cut-off point [10].

### Treatment

Operation: The surgical method, including radical total gastrectomy, radical distal gastrectomy and proximal gastrectomy, was selected depending on the tumor location. Lymph node dissection was performed according to Japanese gastric cancer treatment guidelines (13th edition) [11].

Postoperative adjuvant chemotherapy with 5- fluorouracil (FU) was performed for patients at stage II or above.

Treatment for patients with recurrence: Patients with recurrence after postoperative adjuvant chemotherapy were treated with chemotherapy based on 5-FU plus taxol.

### Statistical analysis

All of the data were analyzed using SPSS 20.0 statistical software. The χ^2^ test was used to compare count data, and the binary logistic regression model was used to analyze independent risk factors for lymph node metastasis. OS and recurrence-free survival were calculated using the Kaplan-Meier method. The log rank test was used to compare survival and recurrence rates. The Cox regression model was used for the multivariate analysis of prognosis. The pros and cons of the prognostic model were compared by combining the area under the receiver operating characteristic (ROC) curve (AUC). *P* < 0.05 was considered a significant difference.

## Results

### Clinicopathologic characteristics

Eighty patients with gMANEC were enrolled in this study from January 2007 to December 2016; 44 were in the NEC group, and 36 were in the AC group. Table 1 shows the clinicopathologic characteristics of the entire cohort (*n* = 80). There was no significant difference between the two groups in terms of male-female ratio, American Society of Anesthesiologists (ASA) score, depth of invasion, lymph node metastasis, TNM stage or Ki-67-positive index, although the NEC group exhibited larger tumor sizes.

**Table 1:** Patient Characteristics

	NEC dominant type	AC dominant type	*P* value
Age(year)			0.071
< 65	16	21	
≥ 65	28	15	
Gender			0.798
Male	33	28	
Female	11	8	
ASA score			0.265
1	20	22	
2	20	10	
≥ 3	4	4	
Tumor size(cm)			0.023
< 5	20	26	
≥ 5	24	10	
Tumor location			0.108
Upper	23	15	
Middle	4	8	
Lower	9	11	
Diffuse	8	2	
T stage			0.202
T1 + T2	5	8	
T3	24	13	
T4	15	15	
N stage			0.095
N0	12	4	
N1	32	32	
TNM stage			0.268
I-II	11	5	
III	33	31	
Operation types			0.145
Total gastrectomy	34	22	
Distal gastrectomy	10	14	
Surgical method			0.631
Open	15	10	
Laparoscopic	29	26	
Complication			0.763
Yes	8	5	
No	36	31	
Ki-67 positive index (%)			0.26
< 60	16	18	
≥ 60	28	18	

### Lymph node metastasis

**Fig. 2:**
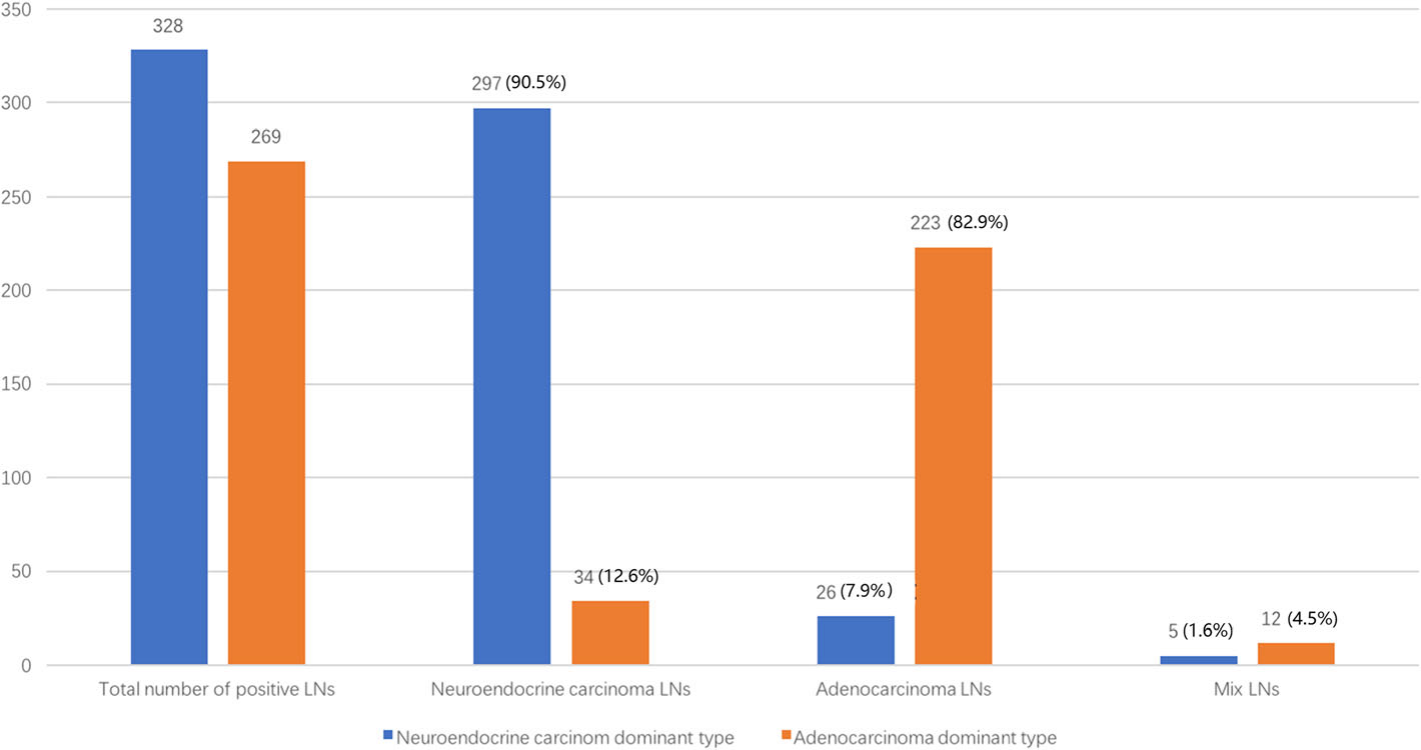
The components of lymph node metastasis in the neuroendocrine carcinoma group and adenocarcinoma group

There were 64 patients of lymph node metastasis and 16 patients without lymph node metastasis. A total of 328 lymph node metastases were identified in 73% (32/44) of NEC group patients, including 297 identified as simple NEC,26 identified as simple AC, and 5 identified as mixed components containing NEC and AC (Fig. 1c). A total of 269 lymph node metastases were identified in 89% (32/36) of the AC group patients, including 223 identified as simple AC, 34 identified as simple NEC, and 12 patients of mixed components containing NEC and AC (Fig. 2). In univariate analysis, T stage (*P* = 0.004) and age < 65 (*P* = 0.023) were associated with lymph node metastasis (Additional file 1: Table S1), and in the multivariate analysis, T stage (*P* = 0.010) and age < 65 (*P* = 0.016) were independent risk factors for lymph node metastasis (Table 2).

**Table 2:** Multivariate analysis of lymph node metastasis in the entire group

	B	SE	Wald	HR	95%CI	*P*
T stage						
T1 + T2						
T3	1.856	0.888	4.369	6.397	1.123–36.455	0.037
T4	3.811	1.285	8.797	45.183	3.642–560.565	0.003
Age < 65	2.137	0.890	5.772	8.478	1.482–48.584	0.016

### Overall survival

**Fig. 3:**
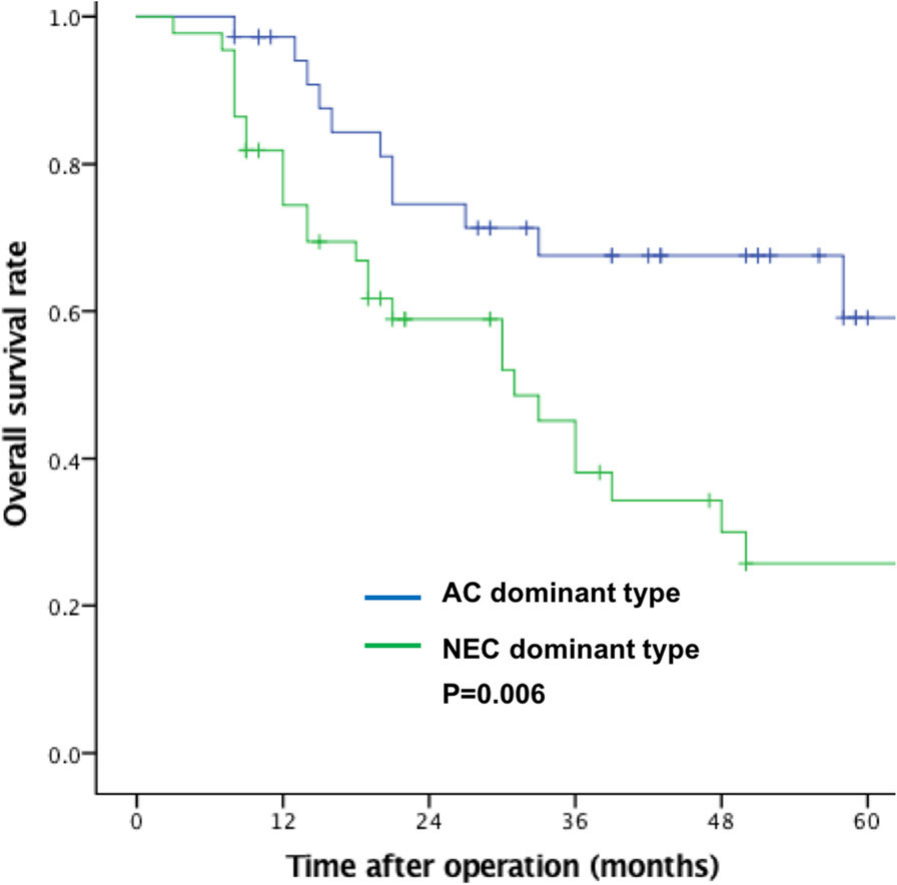
Kaplan-Meier survival curves for overall survival according to types of tumor dominance (NEC: neuroendocrine carcinoma; AC: adenocarcinoma)

The 3-year OS for these 80 patients was 55%, with a median survival of 27 months. The 3-year survival rate of those with NEC-dominant-type tumors (40% vs. 75%, *P* = 0.006) was significantly worse than that of those with AC-dominant-type tumors (Fig. 3). According to the univariate analysis, an NEC-dominant type, a Ki-67-positive index ≥60%, and tumor size ≥5 cm were associated with poor OS. NEC dominance and Ki-67 positivity ≥60% were independent risk factors for OS in the multivariate analysis (Table 3).

**Table 3:** Univariable and multivariable Cox regression analyses of OS

	Univariable			Multivariable		
	*n*	3-year OS (%)	*P* value	HR	95% CI	*P* value
Age(year)			0.738			
< 65	37	54				
≥ 65	43	49				
Gender			0.74			
Male	61	51				
Female	19	56				
ASA score			0.28			
1	42	59				
2	30	49				
≥ 3	8	33				
Tumor size (cm)			0.043	1.537	0.799–2.959	0.198
< 5	46	64				
≥ 5	34	35				
Tumor location			0.288			
Upper	38	50				
Middle	12	75				
Lower	20	51				
Diffuse	10	36				
T stage			0.288			
T1 + T2	13	81				
T3	37	58				
T4	30	36				
N stage			0.327			
N0	16	66				
N1	64	49				
TNM stage			0.235			
I-II	16	65				
III	67	49				
Operation types			0.795			
Total gastrectomy	56	52				
Distal gastrectomy	24	53				
Surgical method			0.084			
Open	25	37				
Laparoscopic	55	60				
Complication			0.647			
Yes	13	62				
No	67	51				
Ki-67positive index (%)			0.002	2.595	1.242–5.425	0.011
< 60	34	73				
≥ 60	46	37				
tumor component in the primary tumor			0.683			
AC higher	34	51				
NEC higher	46	44				
Tumor domination			0.006	2.208	1.078–4.520	0.03
AC dominant type	36	68				
NEC dominant type	44	45				
Adjuvant chemotherapy			0.404			
Yes	50	57				
No	30	39				

### Recurrence-free survival

**Fig. 4:**
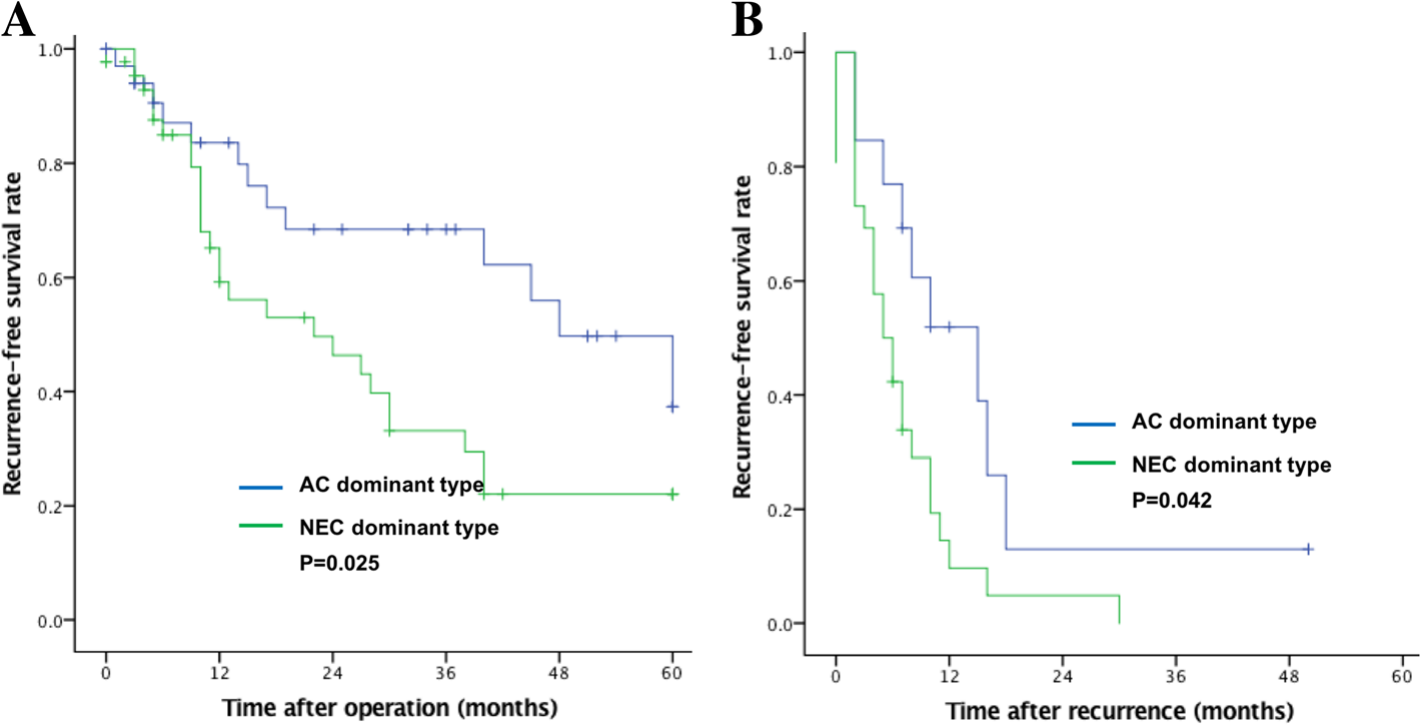
Relationship between types of tumor dominance (NEC: neuroendocrine carcinoma; AC: adenocarcinoma) and prognosis. (**a**) Kaplan-Meier survival curves for recurrence-free survival; (**b**) Kaplan-Meier survival for curves survival time after recurrence

The median follow-up time was 42 months. The 3-year recurrence-free survival was 49%, and the median recurrence-free survival was 19 months. Patients with NEC-dominant-type tumors exhibited decreased recurrence-free survival compared to those with AC-dominant-type tumors (33% vs. 68%, *P* = 0.025), as shown in Fig. 4a. In addition, post-recurrence survival was significantly better for AC-dominant-type patients (10 months vs. 6 months, *P* = 0.042) (Fig. 4b). Among those with NEC-dominant-type tumors, recurrence occurred in 26 patients (60%), consisting of hematogenous recurrence in 17 (65%), peritoneal recurrence in 6 (23%) and local recurrence in 3 (12%). Among the AC-dominant-type patients, recurrence occurred in 13 (36%), including hematogenous recurrence in 7 (54%) and peritoneal recurrence in 6 (46%); this group had no local recurrence. Univariate and multivariate analyses revealed NEC dominance and a Ki-67-positive index ≥60% to be independent risk factors for gMANEC recurrence (Table 4).

**Table 4:** Univariable and multivariable Cox regression analyses of RFS

	Univariable			Multivariable		
	*n*	recurrence	*P* value	HR	95% CI	*P* value
Age(year)			0.663			
< 65	37	22				
≥ 65	43	17				
Gender			0.915			
Male	61	29				
Female	19	10				
ASA score			0.377			
1	42	18				
2	30	17				
≥ 3	8	4				
Tumor size (cm)			0.555			
< 5	46	22				
≥ 5	34	17				
Tumor location			0.119			
Upper	38	18				
Middle	12	3				
Lower	20	11				
Diffuse	10	7				
T stage			0.08			
T1 + T2	13	4				
T3	37	16				
T4	30	19				
N stage			0.182			
N0	16	5				
N1	64	34				
TNM stage			0.249			
I-II	16	6				
III	67	33				
Operation types			0.381			
Total gastrectomy	56	25				
Distal gastrectomy	24	14				
Surgical method			0.162			
Open	25	14				
Laparoscopic	55	25				
Complication			0.081			
Yes	13	7				
No	67	32				
Ki-67positive index (%)			0.026	1.979	1.012–3.870	0.046
< 60	34	14				
≥ 60	46	25				
tumor component in the primary tumor			0.751			
AC higher	34	16				
NEC higher	46	23				
Tumor domination			0.025	1.993	1.016–3.909	0.045
AC dominant type	36	13				
NEC dominant type	44	26				
Adjuvant chemotherapy			0.807			
Yes	50	27				
No	30	12				

### ROC curve prediction of OS and recurrence-free survival

**Fig. 5:**
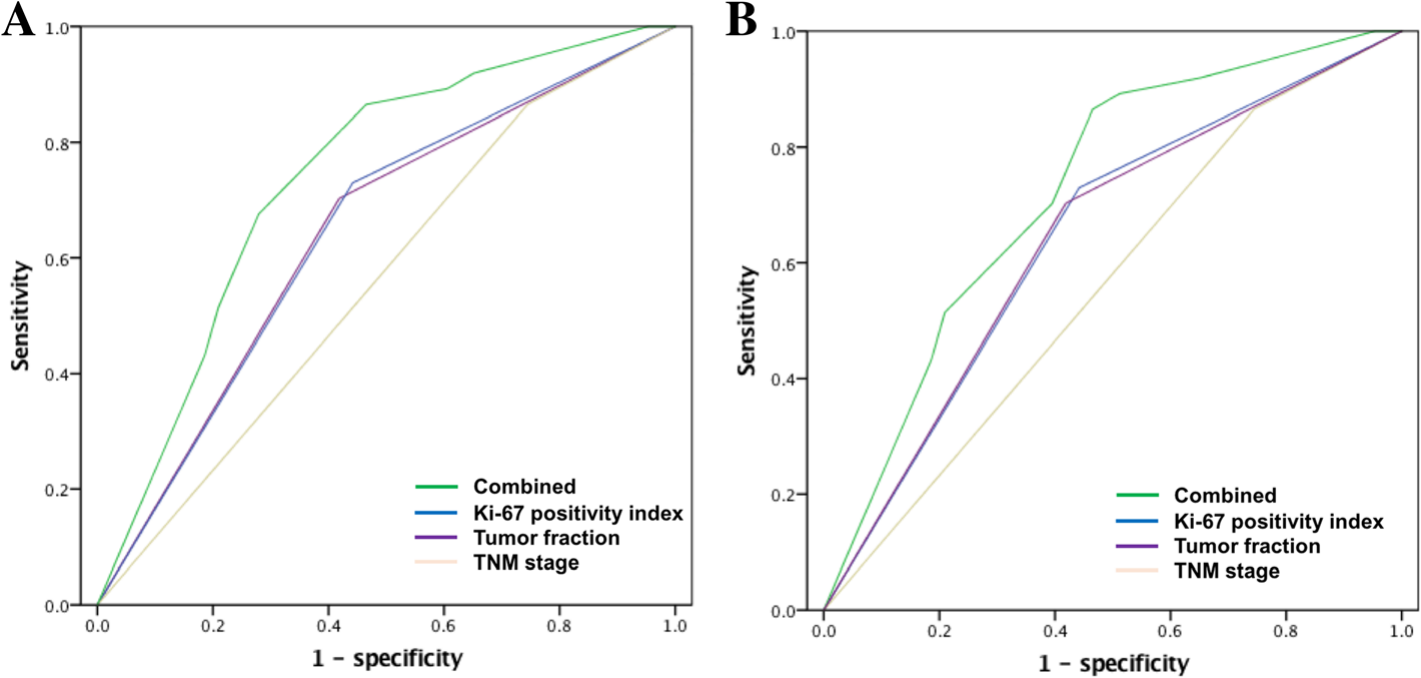
Area under the receiver operating characteristic (ROC) curve of different prognostic factors. (**a**) ROC curve for overall survival; (**b**) ROC curve recurrence-free survival

The discriminative power of the predictive model was expressed as the AUC comparing the degree of similarity between the predicted and actual values. For the 3-year OS, the AUC was 0.574, 0.642 and 0.644 using TNM staging, tumor proportion and the Ki-67-positive index, respectively, with a combined AUC of 0.727 (Fig. 5a). For 3-year recurrence-free survival, the AUC was 0.561, 0.614 and 0.564 using TNM staging, tumor fraction, and Ki-67-positive index, respectively, with a combined AUC of 0.657 (Fig. 5b).

### The impact of tumor dominance on chemotherapy efficacy

**Fig. 6:**
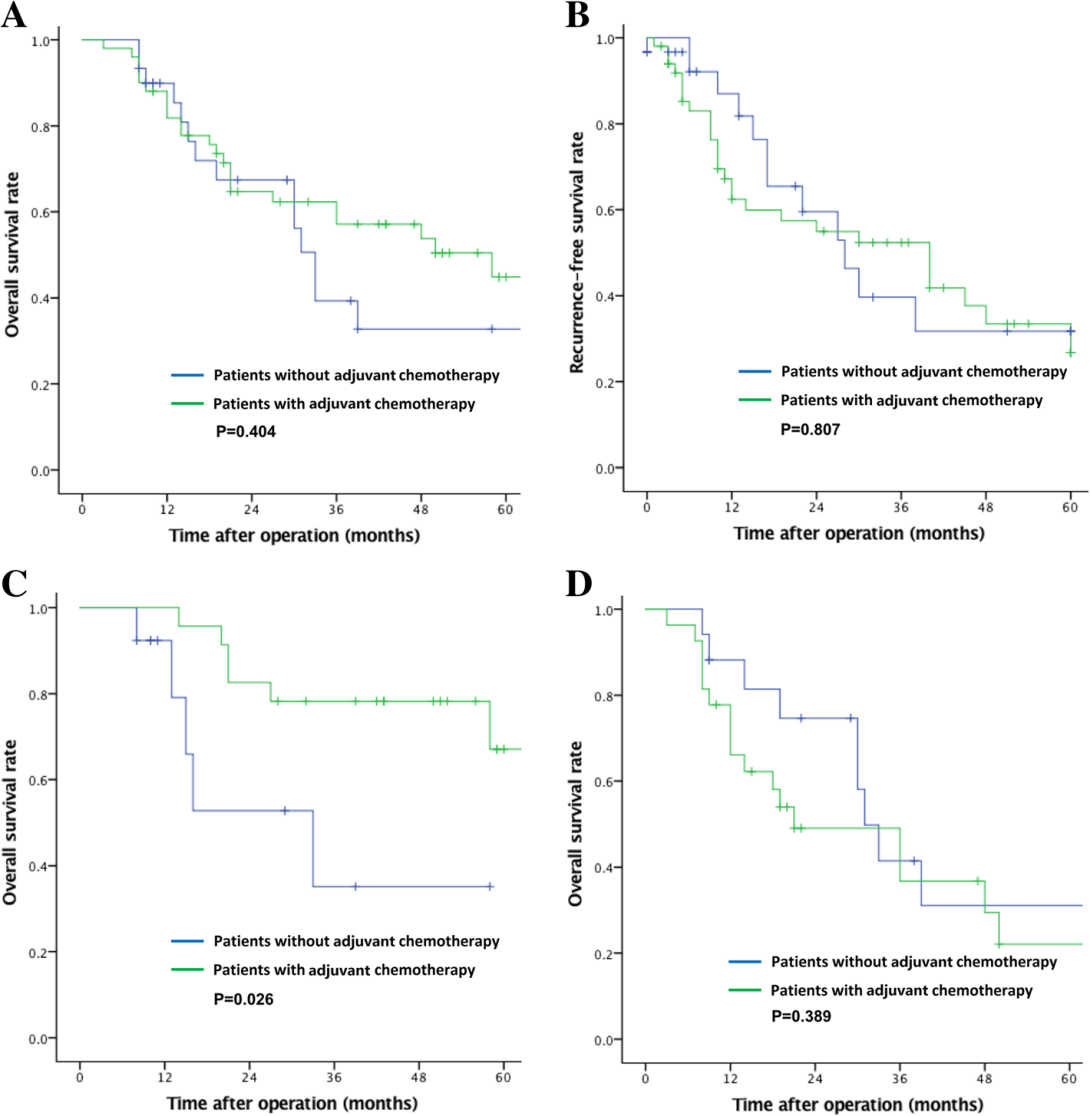
Relationship between adjuvant chemotherapy and prognosis. (**a**) Comparison of overall survival between patients with adjuvant chemotherapy and patients without adjuvant chemotherapy. (**b**) Comparison of recurrence-free survival between patients with adjuvant chemotherapy and patients without adjuvant chemotherapy. (**c**) Comparison of effects of adjuvant chemotherapy and non-chemotherapy on overall survival in adenocarcinoma-dominant-type patients. (**d**) Comparison of effects of adjuvant chemotherapy and non-chemotherapy on overall survival in neuroendocrine carcinoma-dominant-type patients

Fifty patients (60%) received 5-FU-based adjuvant chemotherapy; 30 patients did not. There were no differences in OS and recurrence-free survival between patients that received chemotherapy and those that did not (median OS, 28 vs. 22 months, *P* = 0.404; median recurrence-free survival, 14 vs. 15 months, *P* = 0.807) (Fig. 6a, b). Patients in the AC group treated with adjuvant chemotherapy had a significantly better OS than those that did not receive chemotherapy (median OS, 43 vs. 13 months, *P* = 0.026) (Fig. 6c). In contrast, patients treated with adjuvant chemotherapy in the NEC group had a similar OS to those who did not receive chemotherapy (median OS, 19 vs. 29 months, *P* = 0.398) (Fig. 6d).

## Discussion

There are two hypotheses regarding the origin of gMANEC. The first postulates that AC cells differentiate into neuroendocrine cancer cells [12]; the second posits that monoclonal pluripotent stem cells differentiate into two types of cells [13, 14]. Patients with AC and NEC not only have different prognoses but receive different treatments [15]. Previous research revealed that the clinical features of gMANEC largely depend on the proportion of neuroendocrine cancer components [16, 17]. Fernandes et al. conclude that gMANEC prognosis depends on the more aggressive component [18]. Among 21 gMANEC patients, the proportion of primary tumor components was revealed to be an independent prognostic factor, with a higher rate of NEC component indicating a worse prognosis [19]. In lymph node metastasis-negative (N-) gMANEC, the tumor component present in a higher proportion of the primary tumor suggests the predominant tumor growth. In this study, gMANEC was grouped according to the ratio of primary tumor components in N- patients. For example, if the tumor was more than 50% AC, it was considered the AC-dominant type. In lymph node positive (N+) gMANEC, we found that the composition of the primary tumor was not a factor influencing lymph node metastasis, indicating that tumor components in the primary tumor do not fully reflect malignancy in N+ patients. Therefore, positive lymph nodes in every patient were calculated, and the metastatic components from each positive lymph node were identified. If the lymph node metastases contained more than 50% AC components in N+ patients, the patient was placed in the AC-dominant type group; otherwise, the patient was classified with the NEC-dominant type. For example, if a patient had 10 positive lymph nodes, including 6 simple AC components, 4 simple NEC components, indicating that AC components were 60% (more than 50%); this patient would be placed into the AC group. Thus, gMANEC was divided into two groups: AC and NEC. By comparing OS and recurrence-free survival between these groups, we revealed that this typing method exhibits good prognostic differentiation.

Accurate prognostic prediction is crucial for patients with gMANEC who undergo radical surgery. However, the ability of TNM staging to predict prognosis in those with NEC remains controversial [20, 21]. To our knowledge, whether TNM staging accurately predicts gMANEC prognosis has not been reported. In this study, discriminatory power was limited when using TNM staging to predict gMANEC prognosis (predicted AUC of 0.574 for 3-year OS and 0.561 for 3-year recurrence-free survival). Therefore, we established a prognostic model based on TNM staging combined with the tumor composition ratio and Ki-67 index to provide a reference for identifying patients with a poor prognosis. The model included only two postoperative pathological indicators, which are simple and easily obtained by routine postoperative pathological examination. Overall, the predictive model had good discriminatory ability. The AUCs of the 3-year OS and 3-year recurrence-free survival were 0.727 and 0.657, respectively.

There is no uniform regimen for adjuvant chemotherapy to treat gMANEC. An irinotecan plus cisplatin regimen has yielded good results for NEC [22]. However, Shen et al. used irinotecan plus cisplatin and cisplatin plus etoposide as adjuvant chemotherapy to treat postoperative gMANEC and NEC and showed a similar prognosis between the chemotherapy and non-chemotherapy groups, suggesting that postoperative chemotherapy has no benefit. Accordingly, platinum-based chemotherapy has been recommended as a first-line treatment for gMANEC. In our study, the OS of patients in the AC group treated with adjuvant chemotherapy was significantly better than that of patients that did not receive chemotherapy (median survival time 43 months vs. 13 months, *P* = 0.026), whereas there was no OS advantage for patients in the NEC group that received adjuvant chemotherapy (median survival time 19 months vs. 29 months, *P* = 0.398). Post-recurrence survival among patients in the AC group was also significantly longer than in the NEC group (10 months vs. 6 months, *P* = 0.042). These results indicate that patients in the AC group may benefit from 5-FU-based chemotherapy, which is a popular choice to treat AC.

To our knowledge, this study is the first large-scale study to independently investigate gMANEC prognosis and recurrence. Notably, we classified tumors into the NEC group and the AC group according to gMANEC pathological features. Our results revealed that adjuvant chemotherapy based on 5-FU only benefitted patients in the AC group, suggesting that the clinical modality of gastric AC may be more suitable for AC-dominant-type gMANEC and providing a basis for individualized gMANEC treatment.

This study has several limitations. 1. There is a certain selection bias due to the single-center, retrospective nature of the study. 2. The data are from Eastern countries, with a lack of external verification data, especially from Western countries; thus, multi-center, prospective, large-sample analyses are needed for confirmation of the results.

## Conclusion

Presence of NEC-dominant disease and a Ki-67-positive index ≥60% were significantly associated with worse survival and a higher recurrence rate in gMANEC patients. NEC patients may benefit from receiving gastric adjuvant chemotherapy treatments.

## Additional file

Additional file 1: **Table S1.** Univariate analysis of lymph node metastasis in the entire group. In this univariate analysis, T stage (*P* = 0.004) and age < 65 (*P* = 0.023) were associated with lymph node metastasis. (DOCX 17 kb)
