# Supplementary material for: Prognostic factors for survival after curative resection of gastric mixed adenoneuroendocrine carcinoma: a series of 80 patients
Source: BMC Cancer. 2018 Oct 22;18:1021. doi: 10.1186/s12885-018-4943-z (PMC6198479; doi:10.1186/s12885-018-4943-z)
Supplement: Supplementary file 1 — Table S1. Univariate analysis of lymph node metastasis in the entire group. In this univariate analysis, T stage (P = 0.004) and age < 65 (P = 0.023) were associated with lymph node metastasis. (DOCX 17 kb) [file 12885_2018_4943_MOESM1_ESM.docx]

**Table S1** Univariate analysis of lymph node metastasis in the entire group

|  | N- | N+ | P value |
| --- | --- | --- | --- |
| Age(year) |  |  | 0.023 |
| <65 | 3 | 34 |  |
| ≥65 | 13 | 30 |  |
| Gender |  |  | 1.000 |
| Male | 12 | 49 |  |
| Female | 4 | 15 |  |
| ASA score |  |  | 0.512 |
| 1 | 10 | 32 |  |
| 2 | 4 | 26 |  |
| ≥3 | 2 | 6 |  |
| Tumor size(cm) |  |  | 0.309 |
| <5 | 11 | 35 |  |
| ≥5 | 5 | 29 |  |
| Tumor location |  |  | 0.331 |
| Upper | 6 | 32 |  |
| Middle | 3 | 9 |  |
| Lower | 3 | 17 |  |
| Diffuse | 4 | 6 |  |
| T stage |  |  | 0.004 |
| T1+T2 | 6 | 7 |  |
| T3 | 9 | 28 |  |
| T4 | 1 | 29 |  |
| Ki-67 positive index (%) |  |  | 0.070 |
| <60 | 10 | 24 |  |
| ≥60 | 6 | 40 |  |

N-:lymph node metastasis-negative; N+:lymph node metastasis-positive
